# Septic Infrapatellar Bursitis in an Immunocompromised Female

**DOI:** 10.1155/2018/9086201

**Published:** 2018-06-06

**Authors:** Kenneth Herring, Seth Mathern, Morteza Khodaee

**Affiliations:** Department of Family Medicine, University of Colorado School of Medicine, 3055 Roslyn Street, Denver, CO 80238, USA

## Abstract

Bursitis is a relatively common occurrence that may be caused by traumatic, inflammatory, or infectious processes. Septic bursitis most commonly affects the olecranon and prepatellar bursae. *Staphylococcus aureus* accounts for 80% of all septic bursitis, and most cases affect men and are associated with preceding trauma. We present a case of an 86-year-old female with an atypical septic bursitis involving the infrapatellar bursa. Not only are there very few reported cases of septic infrapatellar bursitis, but also this patient's case is particularly unusual in that she is a female with no preceding trauma who had *Pseudomonas aeruginosa* on aspirate. The case also highlights the diagnostic workup of septic bursitis through imaging modalities and aspiration. This patient had full resolution of her septic bursitis with appropriate IV antibiotics.

## 1. Introduction

The human body contains upwards of 150 bursae, many of which develop after birth [1–4]. These sacs of synovial fluid facilitate motion between various tissue layers of the musculoskeletal system [1, 2, 4–7]. With regard to the knee joint, there are four bursae involved: suprapatellar, prepatellar, deep infrapatellar, and superficial infrapatellar. The main knee bursae are the prepatellar, which overlies the patella, and the superficial infrapatellar bursa, which lies just superior and anterior to the inferior aspect of the patellar tendon [4, 5, 7]. Bursitis, or inflammation of a bursa, is a relatively common occurrence with a wide range of etiologies. Bursitis may be caused by trauma (acute or chronic), inflammation (gout, pseudogout, or rheumatoid arthritis), or infection [1, 6]. There have been many associations drawn between various occupations and their predisposition for specific types of superficial bursitis due to chronic, repetitive microtrauma [1, 6–9].

Septic bursitis occurs when infectious agents—most commonly bacteria—are introduced to the bursa, typically through trauma, cellulitis, or other skin lesions [1, 4, 6, 10]. More than half of septic bursitis cases are preceded by trauma [3, 4, 9, 11]. Due to their anatomical location and relative superficial location, the olecranon and prepatellar bursae are the most common sites of septic bursitis [3, 4]. *Staphylococcus aureus* is the most common bacteria involved in septic bursitis, accounting for 80% of all cases [3, 4, 6, 12, 13]. There have been rare case reports of septic bursitis caused by Prototheca and Mycobacterium species, but this case will focus on a superficial infrapatellar bursitis caused by *Pseudomonas aeruginosa* in an immunocompromised patient [14, 15].

## 2. Case Report

An 86-year-old female with a history of metastatic ovarian cancer presented to the ED with painful bilateral lower extremity edema and a left lateral leg ulceration. Her metastatic ovarian cancer had been diagnosed by malignant pleural effusion five months earlier, and she had completed neoadjuvant chemotherapy with carboplatin and Taxol approximately one week prior to this presentation. She was admitted to the hospital and started on cefazolin for left lower extremity cellulitis on hospital day one.

On admission, plain films and ultrasound did not reveal any evidence of osteomyelitis, fracture, DVT, or abscess to the left lower extremity. On exam, she had 3+ pitting edema below the knee bilaterally as well as chronic venous stasis changes. The patient also had a venous ulcer (approximately 2 cm in diameter) on the anterolateral aspect of the distal third of her left lower leg. At the time of admission, this venous ulcer had some serous weeping but no purulent drainage or fluctuance on examination. Her initial Laboratory Risk Indicator for Necrotizing Fasciitis (LRINEC) score was 4, suggesting a low risk for necrotizing fasciitis; however, on hospital day 3, her CRP began to uptrend and she became febrile. At this point, her antibiotics were switched from cefazolin to vancomycin to cover MRSA.

**Figure 1:**
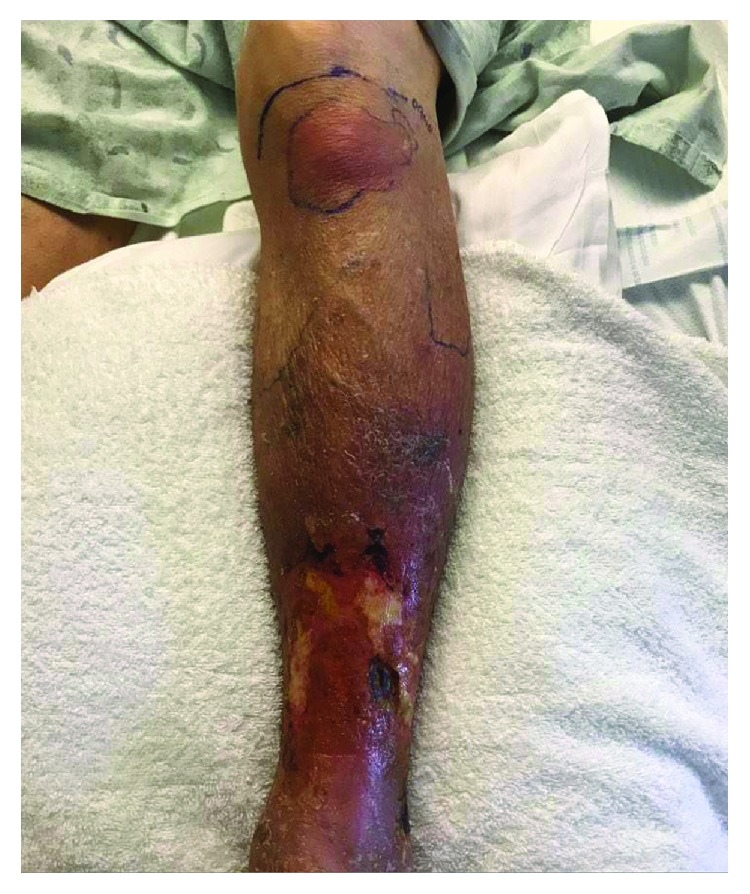
Erythema and swelling anterior and superior to the left tibial tuberosity. Venous ulcer is also noticed on the anterolateral aspect of the distal left lower leg.

**Figure 2:**
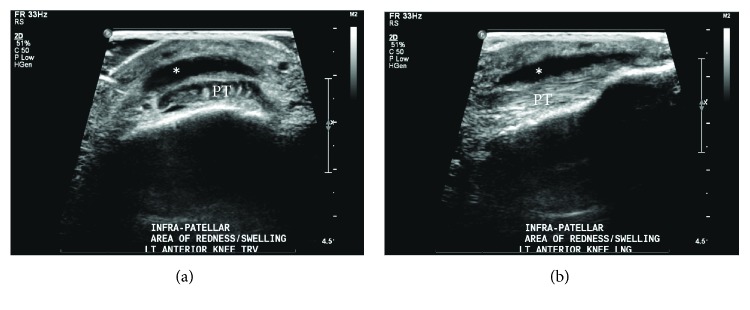
Ultrasound imaging of the left infrapatellar bursa in both cross-sectional (short axis) (a) and longitudinal (long axis) (b) views. ^∗^Infrapatellar bursa is enlarged. The patellar tendon (PT) looks normal.

On hospital day five, the patient was noted to have a new erythematous area over the anterior left knee, inferior to the patella (Figure 1). Ultrasound revealed a small fluid collection superficial to the patellar tendon in the infrapatellar region measuring 3.3 × 2.5 × 0.4 cm (Figure 2). The infrapatellar bursa was aspirated and sent for culture. The patient was started on piperacillin-tazobactam, given the patient's immunocompromised status and subsequent risk for atypical and gram-negative organisms.

An MRI was performed on hospital day seven (this was delayed due to the patient's pacemaker) but did not reveal any evidence of osteomyelitis. The patient was clinically improved after starting piperacillin-tazobactam, and vancomycin was discontinued on hospital day seven. On hospital day eight, aspirate cultures returned with *Pseudomonas aeruginosa*; she was stable for discharge at that time and was sent out with a ten-day course of levofloxacin (culture was pan-sensitive) and close follow-up with infectious disease.

## 3. Discussion

*Pseudomonas aeruginosa* is an uncommon cause of superficial bursitis. Furthermore, there are extremely limited reports of cases of septic infrapatellar bursitis. Only about one-third of all bursitis cases are septic [1]. Of those, 80% are caused by *Staphylococcus aureus*, and the rest are mostly Streptococcal [3, 4]. Septic bursitis is more common in males (80%) [1, 10, 12]. There is some debate as to whether immunocompromised individuals are at increased risk of septic bursitis or simply have more severe presentations. There is data to suggest that up to 50% of septic bursitis cases occur in immunocompromised individuals; however, other data suggests that immunocompromised individuals are at no increased risk [1, 4].

In diagnosing septic bursitis, it is important to differentiate from septic arthritis. Patients presenting with septic bursitis—as opposed to septic arthritis—typically do not have any pain with passive range of motion [9]. Fever is not a strong indicator of septic bursitis, as only 40% of individuals have fever at the time of presentation [3, 9]. Imaging can be an important modality in the diagnosis of septic bursitis. Both ultrasound and MRI have a role in diagnosis—the latter particularly so in evaluating the extent of the infection or evaluating for osteomyelitis [3, 6]. In the absence of bursitis—whether septic or nonseptic—bursae are not visible on ultrasound. It is only in the presence of a disease process that there is enough fluid in the bursa to make it visible with ultrasound imaging [7, 16].

Aspiration of the bursa is an important diagnostic tool. In addition to culturing, the white blood cell count in the aspirate can be useful in discerning septic from nonseptic bursitis. The WBC count in septic bursitis is typically much lower than aspirates in septic arthritis, and it is generally agreed that a WBC count of 1000 to 20,000 is indicative of septic bursitis [1, 3–5, 9, 11, 13]. It is recommended that the aspirate be sent for cell count, culture, gram stain, and crystal analysis [17].

Management of septic bursitis is controversial, but antibiotic coverage is almost universally agreed upon. Unless there are underlying risk factors, first-line therapy involves either a first-generation cephalosporin (e.g., cefazolin) or a penicillinase-resistant penicillin (e.g., oxacillin) intravenously for Staphylococcal and Streptococcal coverage [1, 3–6, 17]. The duration of therapy is less agreed upon, ranging anywhere from ten days to four weeks with most recommendations closer to two weeks [4, 6, 11, 13]. Antibiotics can be narrowed based on culture results; however, immunocompromised patients may require seven to ten days of IV antibiotics [1]. In addition to antibiotics, many treatment recommendations include aspiration and drainage of the affected bursa [4, 11, 13]. Aspiration can alleviate symptoms and reduce the bacterial burden [17]. There is some data to suggest that there is no treatment difference in drained or nondrained bursae [3]. In severe, recurrent, or refractory cases, surgical bursectomy may be warranted [3–6, 10, 13].

The patient in this case is an atypical presentation of septic bursitis for several reasons. As mentioned above, there are very few reported cases of septic infrapatellar bursitis. Furthermore, as mentioned above, bursitis disproportionately affects males (80%), it is largely caused by *Staphylococcus aureus* (80%), and most cases are preceded by trauma (>50%). This patient not only had involvement of a very uncommon bursa, but she had no preceding trauma and grew Pseudomonas from aspiration. Unfortunately, her aspirate was not sent for cell counts but was appropriately placed on piperacillin-tazobactam given her risk for atypical and gram-negative organisms. The most likely source of this patient's septic bursitis was the ulceration on the anterolateral aspect of her left distal leg. The authors are unable to determine the timing of the pseudomonal infection or whether the bacteria were present in the ulcer prior to admission. Involvement of the infrapatellar bursa, however, most likely arose from the lower leg cellulitis and subsequent spread from nearby infected tissues, as hematogenous spread is rare. The patient has had full resolution of her infrapatellar bursitis with no recurrence one month out from her initial presentation.
